# Hybrid phantom for lung CT: Design and validation

**DOI:** 10.1002/mp.17990

**Published:** 2025-08-08

**Authors:** Paulo Roberto Costa, Gisell Ruiz Boiset, Elsa Bifano Pimenta, Raphael Moratta Vieira Rocha, Raissa Aline Santos Moura, Wagner Henrique Marques, Luuk J. Oostveen, Bram Geurts, Marcio Valente Yamada Sawamura, Denise Yanikian Nersissian, Elisabeth Mateus Yoshimura, Ioannis Sechopoulos

**Affiliations:** ^1^ Instituto de Física Universidade de São Paulo (USP) São Paulo São Paulo Brazil; ^2^ Department of Medical Imaging Radboud University Medical Center Nijmegen The Netherland; ^3^ Departamento de Radiologia e Oncologia Faculdade de Medicina, Universidade de São Paulo (USP) São Paulo São Paulo Brazil; ^4^ Hospital das Clinicas HCFMUSP, Faculdade de Medicina Universidade de São Paulo (USP) São Paulo São Paulo Brazil

**Keywords:** 3D printing, anthropomorphic phantom, LDCT, lung imaging

## Abstract

**Background:**

CT lung imaging protocols need to be optimized. This claim is especially important due to the possible introduction of low‐dose CT (LDCT) for lung cancer screening. Given the incorporation of non‐linear reconstructions and post‐processing, the use of phantoms that consider task‐based evaluation is needed. This is also true for acceptance and continuous QC use.

**Purpose:**

To present and validate a lung‐CT hybrid phantom composed of two setups, one for task‐based image quality metrics and the other anthropomorphic.

**Methods:**

The task‐based metrics setup was based on the well‐known Mercury phantom and the anthropomorphic setup named Freddie (from **F**igure of Me**r**it P**e**rformance evaluation of **D**etectability in **D**iagnostic CT **I**maging **E**quipment) was designed with the same basic dimensions of the Mercury phantom, but including pieces and materials for mimicking chest structures, such as tracheobronchial tree and lung parenchyma. This setup allows the inclusion of pieces of different sizes to mimic ground‐glass opacities, and sub‐solid and solid lung nodules. The validation of the phantom adopted three methods: comparative evaluation of the attenuation properties and the corresponding Hounsfield Units (HU) values of the selected materials; image assessment according to five chest radiologists and eight non‐radiologists’ observations (reader study), and measurement of task‐based metrics. Images of both setups were acquired using two clinical thorax protocols, both using automatic tube current modulation (TCM). Two x‐ray filter combinations were adopted. The images were reconstructed using a deep learning‐based algorithm.

**Results:**

The agreement of nominal and observed HU values in the task‐based setup was within 15%, except for three (TangoBlack+, VeroClear, and HIPS) of the materials employed in the phantom construction, at some beam energies. In the reader study, synthetic solid nodules printed in VeroClear received average Likert scores 4.0 (range 3.0–4.0) from radiologists and 3 (range 2.6–3.8) from non‐radiologists, printed in TangoBlack+ received an average Likert score of 3.9 (range 3.8–4.2) from radiologists and 4.0 (range 3.8–4.4) from non‐radiologists, while those printed in HIPS scored an average Likert of 3.8 (range 3.3–3.9) from radiologists and 3.3 (range 3.1–3.3) from non‐radiologists. The synthetic ground‐glass opacities (GGO) nodules manufactured in EVA received an average Likert score of 3.8 (range 2.8–4.6) from radiologists and 4.3 (range 3.6–4.8) from non‐radiologists. The task‐based setup demonstrated detectability index variations across protocols influenced by the dose levels, voltage, and x‐ray beam filtration used.

**Conclusions:**

The novelty of the proposed design is concentrated on the possibility of associating the response of the task‐based setup (Mercury) with a patient‐based setup (Freddie) in a unique phantom. This hybrid design enhances the potential to apply the detectability index for optimizing CT protocols in clinical scenarios.

## 1 INTRODUCTION

Optimizing the balance between detection sensitivity and radiation‐associated risks is crucial in low‐dose chest CT for lung cancer screening and nodule follow‐up.^1^, ^2^, ^3^, ^4^⁠ Iterative reconstruction (IR) has been widely adopted clinically as an option for improving image quality by maintaining or reducing noise and doses compared to filtered backprojection (FBP). More recently, deep learning reconstruction (DLR) algorithms have been adopted to improve traditional image methods with excellent results for brain,^5^ abdomen,^6^⁠ and lung.^7^, ^8^ ^⁠^

These contemporary reconstruction algorithms, which are more sophisticated and computationally intensive compared to traditional analytical ones^⁠^,^9^, ^10^, ^11^ violate the well‐known relation between noise and dose. This results in the inadequacy of traditional image quality (IQ) metrics^⁠^.^12^, ^13^ Additionally, tradeoffs between image sharpness, artifact suppression, and noise reduction can severely compromise the accuracy of these IQ metrics.^14^ ^⁠^ IQ metrics, such as noise power spectrum (NPS) and task‐based modulation transfer function (TTF) are useful in order to evaluate the noise and spatial resolution characteristics of phantom images.^15^, ^16^ ^⁠^ The AAPM Task Group Report 233^17^, ^18^ ^⁠^ presents different methods for the establishment of Figures of Merit (FOMs) related to modern CT techniques.^19^, ^20^ This report also exemplifies the use of different phantom designs to quantitatively assess image quality by these metrics and their combination, resulting in the detectability index.

However, the association between the detectability index and the capabilities of an observer to identify relevant clinical findings in CT images is still not fully established.^21^⁠ The current phantom designs assume that the capabilities of CT equipment and protocols are well assessed by simple, uniform, and non‐anatomically shaped low‐contrast structures for simulations of lesion detection in patients.^22^, ^23^ ^⁠^ The adoption of the detectability index for protocol optimization purposes in realistic clinical situations is limited, since it depends on images of patient cohorts with similar anatomical characteristics imaged using different protocols and reconstruction methods. Therefore, hybrid anthropomorphic‐physical phantoms combining capabilities to evaluate task‐based quantities and detection of patient‐mimicking structures are convenient for protocol optimization purposes.

In the special case of chest CT, the development of anthropomorphic phantoms integrating the attenuation properties of the lung with clinically relevant structures, such as lung nodules, is receiving attention in recent years. A detectability study comparing the performance of two types of phantoms was published by Cozelmann et al.^24^ ^⁠^ The authors concluded that low‐contrast lesions imaged using uniform background phantoms are not representative of lesions imaged using anatomically designed phantoms. In a protocol optimization approach, these differences can affect predictions of system performance. Solutions for the design and 3D printing of lung nodules were proposed by Hatamikia et al.^25^, ^26^ ^⁠^ and Rinaldi et al.^27^ ^⁠^ In particular, lung lesions of various shapes, sizes, heterogeneities, and radiodensities were modeled and printed recently by Hong et al.^28^ ^⁠^ and Hatamikia et al.^26^ ^⁠^ However, as emphasized by Filippou and Tsoumpas,^29^ ^⁠^ there are limitations on the printing materials used, since they do not adequately mimic the x‐ray attenuation properties of the corresponding tissues.^30^⁠ More recently, Shunhavanich et al.^31^ ^⁠^ developed a 3D printed phantom with a high quantity of sub‐millimeter inserts using the PixelPrint technique.^32^, ^33^, ^34^⁠ This phantom was designed to investigate detectability using a non‐prewhitening (NPW) model observer across different reconstruction algorithms and exposure levels.

This work presents the construction and validation of a phantom designed to support lung CT protocol optimization. The phantom combines both task‐based image quality metrics and anthropomorphic structures, enabling the evaluation of task‐based image quality metrics as well as clinical detectability of lesions. The task‐based metrics setup is based on the well‐established Mercury phantom^35^, ^36^ ^⁠^ and therefore includes the ability to evaluate the tube current modulation (TCM) response of CT systems.

## 2 MATERIALS AND METHODS

### 2.1 Phantom design

The hybrid phantom proposed in the present work is composed of two different setups. The first setup (task‐based metrics) is a homemade version of the Mercury phantom.^35^, ^36^ Its main body with modules of five diameters (12 –37 cm) was built using ultra‐high molecular weight polyethylene (UHMW). Each module includes one UHMW homogeneous section for NPS evaluation and a section with cylindrical inserts of different materials for TTF measurements.

The second setup (anthropomorphic), named Freddie (from **F**igure of Me**r**it P**e**rformance evaluation of **D**etectability in **D**iagnostic CT **I**maging **E**quipment), includes patient‐mimicking modules of the same diameter of the original Mercury. It is designed to accommodate 3D printed structures that mimic the tracheobronchial tree and lung nodules. A specific material for mimicking the attenuation of the pulmonary parenchyma, a 3D printed vertebra, and other attenuating structures are also included.

**FIGURE 1:**
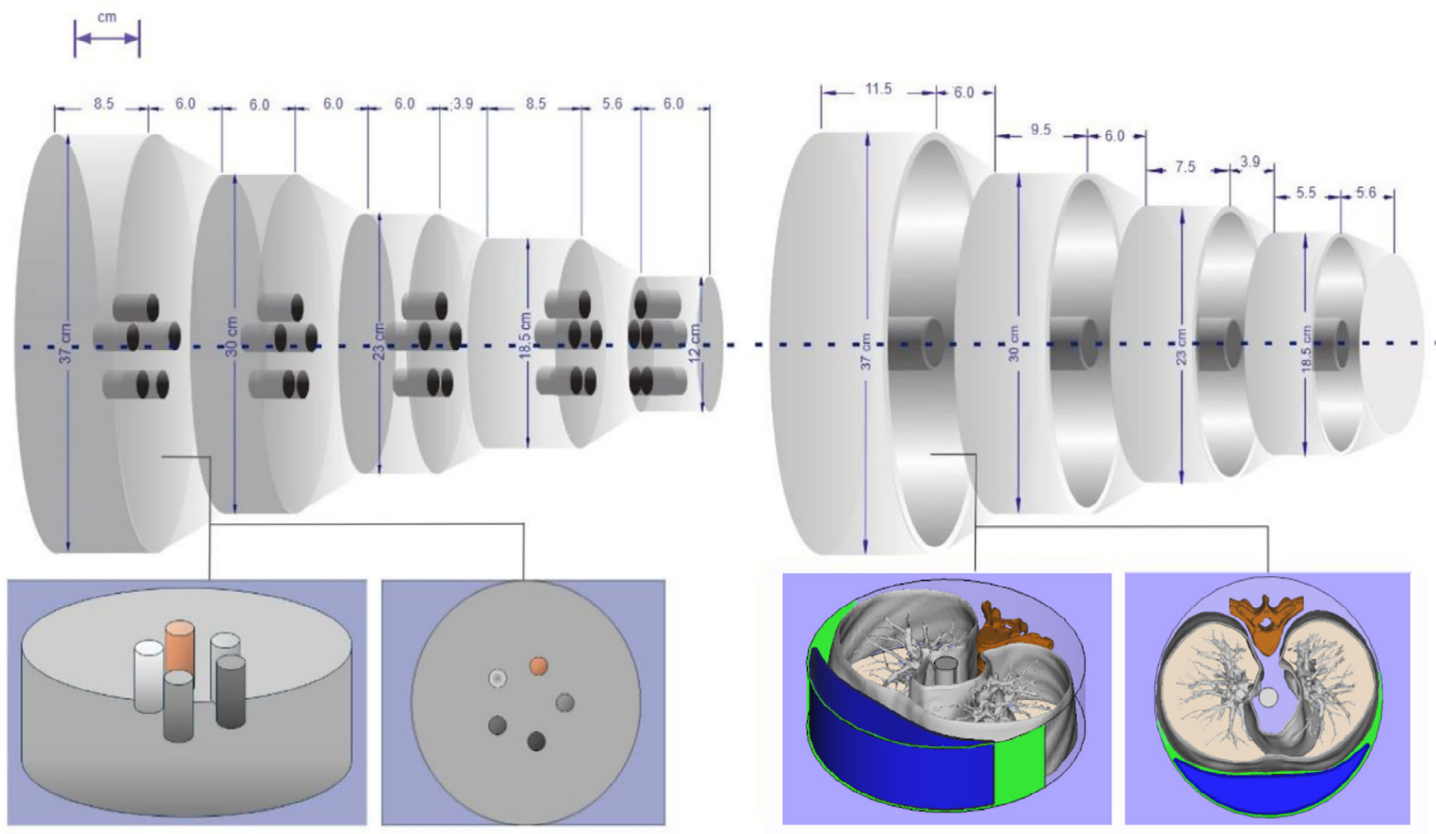
Schematic drawings of the Freddie‐Mercury phantom. The left setup represents the Mercury phantom, considering the original geometric design. The right setup represents the Freddie option, with lung inserts, soft tissue compensation (in blue), vertebrae (in red), and lung equivalent foam (in light beige). To prevent movement of the structures, a loofah sponge was placed between the lung and soft tissue compensation (in green).

Figure 1 shows the basic schematic design of the Freddie–Mercury phantom. Both setups may use the same UHMW central axis and supporting/alignment base. Table 1 presents materials and manufacture techniques selected for the phantom construction.

**TABLE 1:** Materials and manufacturing techniques were selected for the phantom construction.

Phantom module	Material	Material type	Manufacturer	Manufacturing technique	Equipment model	Intended use	Appearance
**Freddie–Mercury**	UHMW	Polymer	MP^a^	Machining	–	Main body, axis, and support/alignment system	Solid (Mercury) and hollow (Freddie) cylinders and rods
**Mercury**	PMMA	Polymer	MP^a^	Machining	–	TTF Inserts	2.5 × 3.0 cm^2^ cylinders
	Polypropylene	Filament	GTMax^b^	FDM^1^	Core H5	TTF Inserts	
	Nylon	Filament	GTMax^b^	FDM^1^	Core H5	TTF Inserts	
	HIPS	Filament	GTMax^b^	FDM^1^	Core H5	TTF Inserts	
	Polyurethane	Polymer	MP^a^	Machining	–	TTF Inserts	
**Freddie**	VeroBlue	Resin	Stratasys^c^	PolyJet^2^	Objet Connex 350	Lung stem and parenchyma	Anthropomorphic structures
	TangoBlack+	Resin	Stratasys^c^	PolyJet^2^	Objet Connex 350	Lung nodules	
	VeroClear	Resin	Stratasys^c^	PolyJet^2^	Objet Connex 350	Lung nodules	
	HIPS	Filament	GTMax^b^	FDM^1^	Core H5	Lung nodules	
	EVA	Foam	Make+^d^	Hand‐molding	–	Lung nodules	
	Packing protection foam	Chips	Vendrig P. BV^e^	–	–	Lung parenchyma	

^1^ Fused Deposition Modeling (FDM).

^2^ Multi‐material Jetting (PolyJet).

^a^ Macedo Plasticos, São Paulo, Brazil (https://macedoplasticos.com.br/).

^b^ GTMax 3D, São Paulo, Brazil (https://www.gtmax3d.com.br/).

^c^ Stratasys Inc, USA (https://www.stratasys.com/en/).

^d^ Make+, Florianópolis, Brazil (https://www.produtosmakemais.com.br/).

^e^ Vendrig Packaging BV, Montfoort, Netherlands (https://vendrigpackaging.com/).

### 2.2 Mercury (task‐based metrics) setup design

The five cylindrical modules have dimensions (length x diameter) of 8.5 cm × 37.0 cm, 6.0 cm × 30.0 cm, 6.0 cm × 23.0 cm, 8.5 cm × 18.5 cm, and 6.0 cm × 12.0 cm. Intermediate connection modules were constructed considering the two diameters of the adjacent cylindrical modes and a conical profile sector (Figure 1). In each cylindrical module, five holes of 3.0 cm length and 2.5 cm diameter were drilled to accommodate rods of different materials for TTF evaluations. The rods included in this Mercury configuration were polypropylene, polymethyl methacrylate (PMMA), high‐impact polystyrene (HIPS), nylon, and polyurethane. These materials are distinct from previous setups of the Mercury phantom^35^ ^⁠^ and were chosen based on their relatively low contrast response when imaged using lung CT protocols. This choice may allow an association between the resulting detectability indexes and observational experiments using the anthropomorphic configuration (Freddie, described in the next section).

**FIGURE 2:**
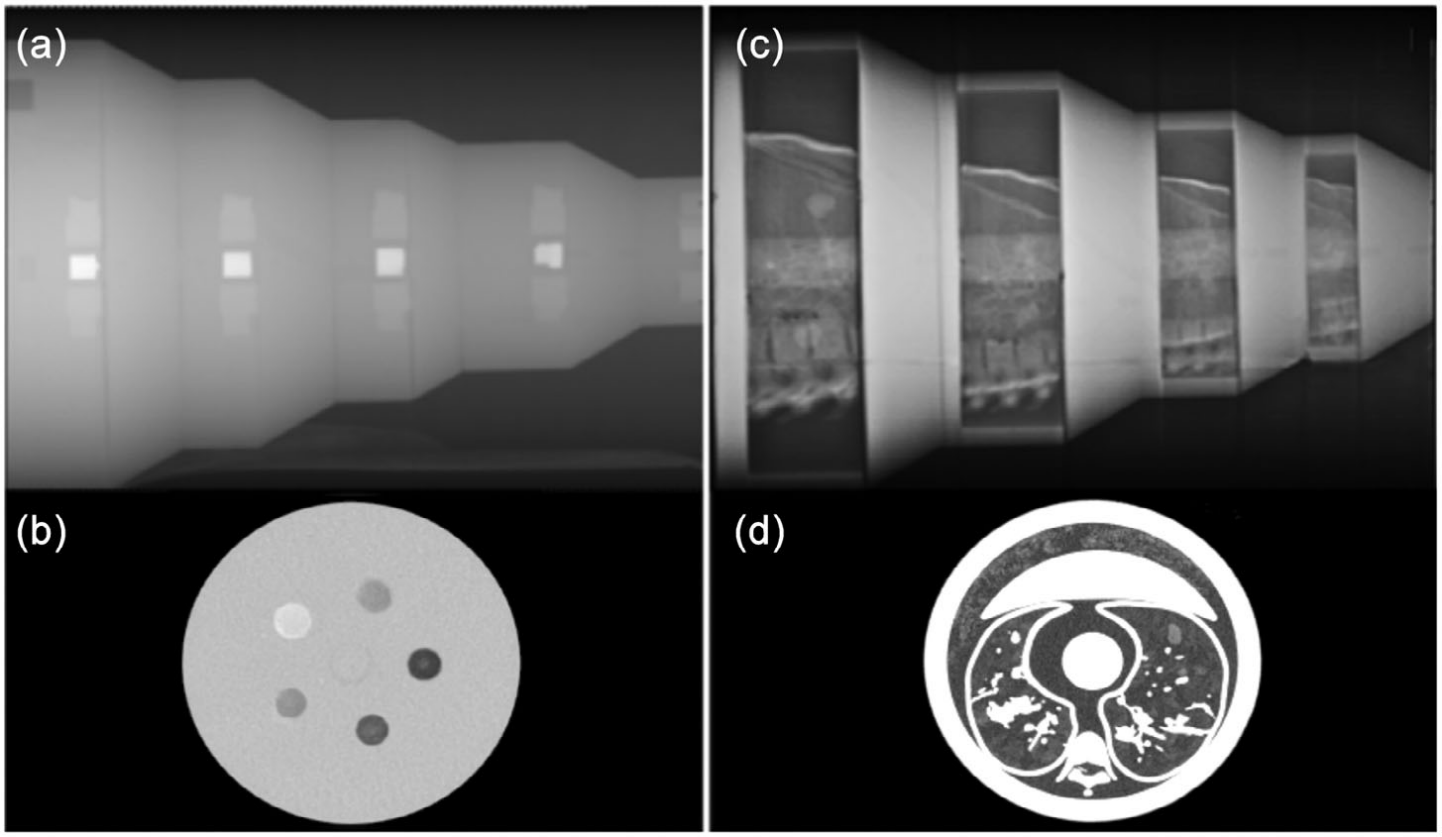
Mercury (physical) phantom in the sagittal (a) and (b) and axial planes Freddie (anthropomorphic) phantom in the sagittal (c) and axial planes (d), respectively.

In addition to measuring the TTFs using these materials, the Mercury modules contain a uniform region of UHMW used for NPS evaluation (Figure 2). These two metrics were used to calculate the detectability index associated with each contrast level studied. Details on how these detectability indices were calculated are presented in Section 2.4.3.

### 2.3 Freddie (anthropomorphic) setup design

#### 2.3.1 Basic lung structure

The anthropomorphic part of the developed phantom is intended to mimic the attenuation properties of normal lung tissues and also to incorporate synthetic lung nodules. It is important to emphasize that the aim was not to replicate CT lung images identical to those of patients. Instead, the focus was on reproducing the attenuation characteristics of the human chest at varying diameters. Therefore, four sections of a Mercury phantom were machined to create cavities to accommodate the 3D printed lung‐mimicking pieces described in the next paragraphs.

**FIGURE 3:**
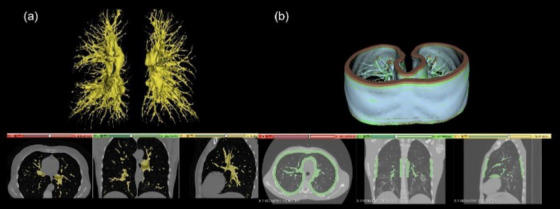
Examples of segmentation of the lung tissue of interest: (a) tracheobronchial tree and (b) lung pleura.

**FIGURE 4:**
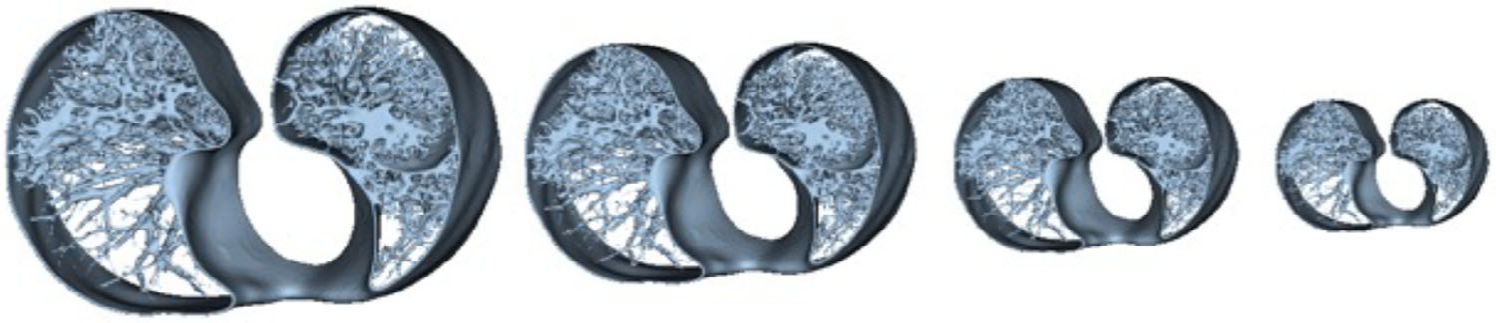
Adjusted slice segmentations for each Freddie module, from largest to smallest.

**FIGURE 5:**
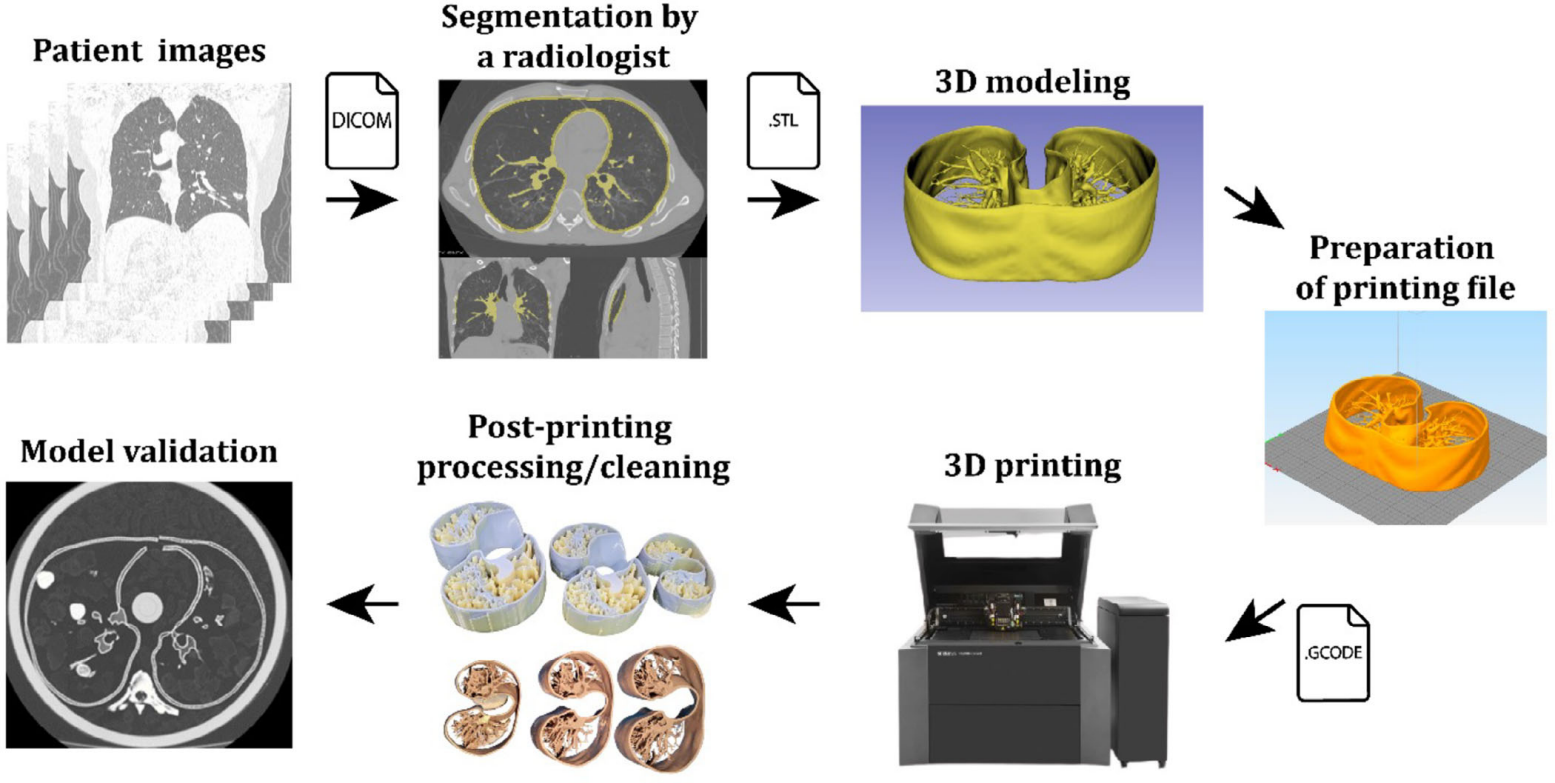
Steps adopted on the 3D modeling of the Freddie setup of the developed phantom, from patient images to the model validation using CT images. CT, computed tomography.

The simplified tracheobronchial tree and lung parenchyma anatomy were modeled for 3D printing compatibility. CT images of a patient were selected by a chest radiologist (MVYS) from the patient database of the Hospital das Clínicas, Faculdade de Medicina, Universidade de São Paulo (HCFMUSP), São Paulo, Brazil.^1^ The images were anonymized, and the lung tissue components of the images were segmented using an open‐source software (3D Slicer, version 5.6.2, Slicer Community)^37^ (Figure 3). These segmentations were then rescaled to match the internal diameters of the four Freddie sections (Figure 4) and converted into G‐CODE files using Simplify3D® (www.simplify3d.com) software. Figure 5 shows the steps for modeling the Freddie setup.

The PolyJet VeroBlue photopolymer (Stratasys Inc., USA) was selected to produce models of the tracheobronchial tree and lung pleura with more realistic details and attenuation properties. This choice was based on the proximity of the VeroBlue photopolymer Hounsfield Units (HU), 88 ± 8 HU at 120 kV, to those of pulmonary structures.^38^ After various attempts with different materials (example: EPS, Greek sponge, natural sponge, and air), foam chips used for packing protection were considered the best choice to represent lung parenchyma by two chest radiologists (MVYS and BG) by judging the CT scans of the anthropomorphic setup. These chips were broken into smaller pieces (approximately 10 × 2  mm^2^) and positioned in the aerial parts of the Freddie setup.

For evaluating the adequacy of this material, 10 HU measurements of the Freddie parenchyma‐mimicking material were collected across the slices of the phantom images using 25 mm^2^ ROIs. The HU values were analyzed using imQuest, version 7.2 (Duke University, USA), and converted to density (g/cm^3^) using the standard equation ρ=(HU/1000)+1, assuming water at 1 g/cm^3^ (0 HU).

#### 2.3.2 Design of the lung nodules

**FIGURE 6:**
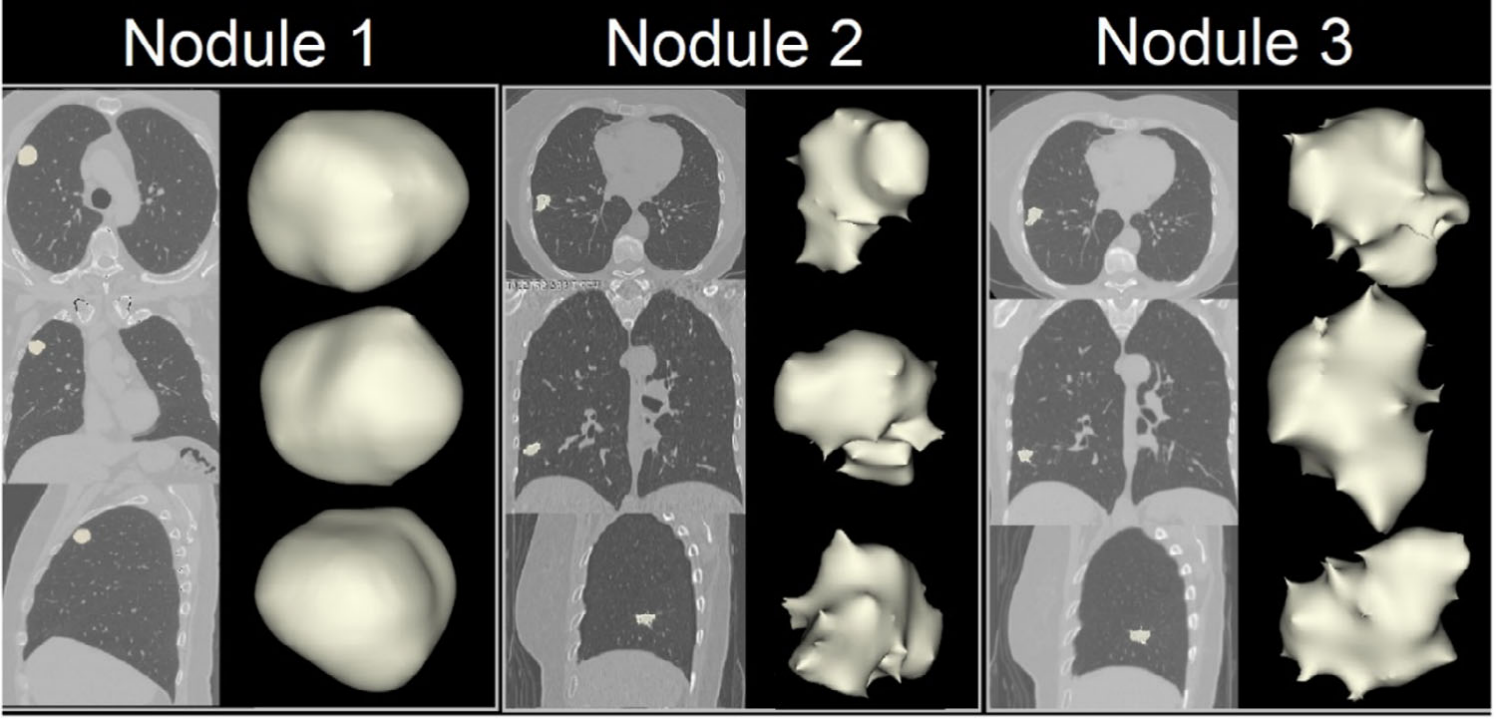
Lung nodules located in the patient's lung CT images and corresponding segmentations are to be printed. CT, computed tomography.

To introduce structures to mimic lung nodules into the Freddie phantom, three patient images were selected, and solid lung nodules were identified by a radiologist (MVYS). These nodule images were segmented and modeled to be printed using the same methods described above (Figure 6). They were also rescaled to be printed in eight different sizes, corresponding to nominal volumes of 5 mm^3^ (XXS), 24 mm^3^ (XS), 68 mm^3^ (S), 154 mm^3^ (M), 1231 mm^3^ (L), 1882 mm^3^ (XL) and 6829 mm^3^ (XXL), respectively. At least one synthetic nodule of each of the three morphologies presented in Figure 6, was printed considering one of these volumes. These synthetic solid nodules were printed in VeroClear, TangoBlack+, and HIPS. Additionally, synthetic nodules using ethylene vinyl acetate (EVA) were hand‐molded in ten different sizes and formats in order to represent ground‐glass opacities (GGOs). Finally, combined structures of EVA and printed solid nodules (XXS‐ and XS‐sizes) were produced to represent subsolid nodules.

To verify the actual volumes of all nodules after printing, they were imaged using a µCT system model U‐SPECT6CThr (MILabs B.V., Netherlands) with a 25 µm focal spot size and 30 µm^3^ voxel size. The ultra‐high resolution µCT images of each nodule were segmented using the 3D Slicer software, and their volumes were calculated as the number of voxels estimated by the segmentation times the volume of each voxel. A similar approach was recently adopted by D'hondt et al.^39^ The type A uncertainties associated with the measurements were determined by calculating the standard deviation of volumes resulting from five independent segmentations of each nodule. Additionally, type B uncertainty was estimated by calculating the number of voxels on the surface of the nodule and determining the corresponding volume, half of this value was considered to account for the uncertainty at the boundary. Although Type A uncertainty inherently includes segmentation variability, which may be influenced by voxel resolution, Type B uncertainty was treated separately to explicitly account for systematic effects that are not purely stochastic. A Pearson correlation test revealed a statistically significant positive correlation (*p* < 0.05) between Type A and Type B uncertainties, with a correlation coefficient of 0.7. Therefore, the combined uncertainty was calculated as the square root of the sum of the squares of Type A and Type B uncertainties, plus twice the product of the correlation coefficient, Type A uncertainty, and Type B uncertainty.

#### 2.3.3 Design of other attenuation anthropomorphic structures

**FIGURE 7:**
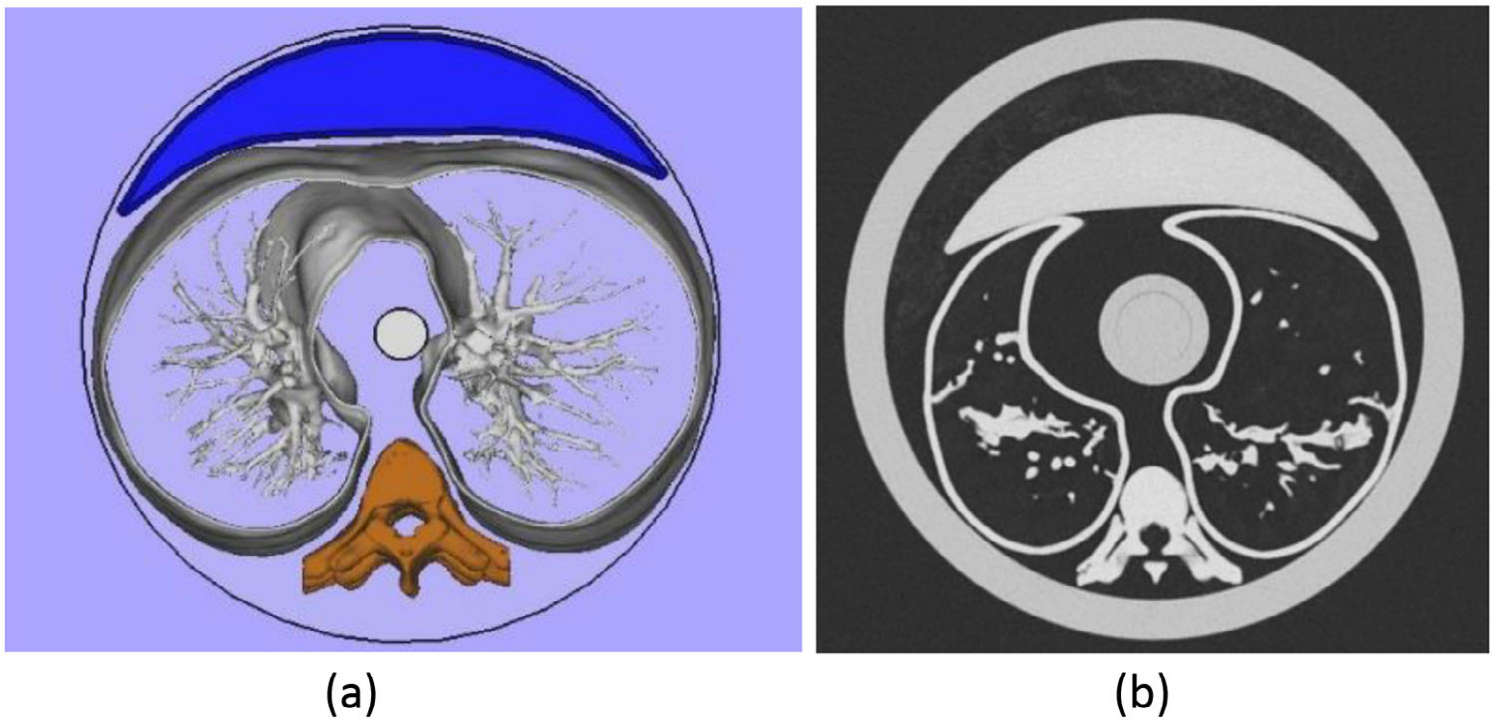
Basic structures of the Freddie phantom modules. The blue structure presented in (a) is printed in ABS and filled using the ReH2O resin.^41^, ^42^ The orange part printed in PETG in (a) corresponds to the thoracic column of the same patient. These structures are introduced into the phantom to optimize the TCM response. In (b), the result of introducing these structures can be observed in a phantom image. PETG, polyethylene terephthalate; TCM, tube current modulation.

To approximately match phantom and human chest attenuation in order to adequate the CT systems TCM response, soft‐tissue and thoracic column compensation pieces were designed for each Freddie module diameter. Half‐moon shaped soft‐tissue pieces were printed in ABS and filled with the ReH2O resin developed using a previously published method^⁠^.^40^, ^41^ These pieces were incorporated in the anterior part of each section to simulate the attenuation of the pectoral muscles (Figure 7a). Additionally, a patient's thoracic column images were segmented, modeled, and 3D‐printed in polyethylene terephthalate (PETG) using a GTMax3D Core H5 (GTMax 3D, São Paulo, Brazil) printer. These pieces were located in the position corresponding to a real patient. An axial scan of one of the fully assembled modules is shown in Figure 7b.

### 2.4 Freddie–Mercury phantom characterization and validation

The characterization and validation of the Freddie–Mercury phantom was performed in three steps:

1. Material attenuation characterization (Freddie–Mercury): The attenuation properties of the selected phantom materials at the effective energies of interest for CT chest examinations were investigated.
2. Observers assessment validation (Freddie): Freddie images were presented to chest radiologists and non‐radiologists observers to identify the best materials for solid/semi‐solid nodules, ground‐glass opacities, and lung parenchyma. Accuracy and precision of the nodule volumes were also estimated.
3. Task‐based metrics characterization (Mercury): Images from the Mercury setup with low‐contrast inserts were used to calculate task‐based metrics, evaluating the setup's response consistency.

#### 2.4.1 Material attenuation characterization

Different polymers and resins were studied by assessing their linear attenuation coefficients and the expected HU values during a pre‐selection stage of the materials adopted for the phantom construction. Only the materials that resulted in the expected HU and contrast ranges compared to chest patient CT images were used in the construction of the prototype. The adopted adequacy criterion was that the expected contrast produced should not differ more than 40% from the patient contrast in the same anatomical region.

For the phantom validation, images of both setups were acquired using a clinical CT scanner (Aquilion One PRISM Edition, Canon Medical Systems, Otawara, Japan). Two clinical thorax protocols were used (Table 2): CT thorax (CTDI_vol_ = 2 and 3 mGy) and thorax low‐dose (CTDI_vol_ = 0.4 mGy), both using automatic TCM. The CT Thorax protocol is considered for lung cancer screening studies, while the low‐dose thorax protocol is used for patient follow‐up. The image reconstruction algorithm was AiCE (Advanced intelligent Clear‐IQ Engine) Lung standard. AiCE is a deep learning‐based reconstruction technique for CT images that enhances image quality by utilizing a deep convolutional neural network. Two x‐ray filter combinations were used: aluminum‐copper (Al+Cu) and aluminum‐silver (Al+Ag). The following acquisition settings were kept constant for both protocols: slice thickness, 0.5 mm; pitch, 0.813; rotation time, 0.275 s; beam collimation, 0.5 × 80 mm. The noise level (SD) of the automatic TCM was set to 15 for the CT thorax protocol and to 35 for the thorax low‐dose protocol. These settings are applied for a 5 mm reconstruction, as used clinically in our institution.

**TABLE 2:** Protocols adopted for testing and validation of the developed phantom.

Reference code	Protocol	Noise level (SD)	Voltage (kV)	Beam filter	Tube current range (mA)	CTDI_vol_ (mGy)
P1	**CT Thorax**	15	100	Al + Cu	80–700	3
P2			120	Al + Ag		2
P3				Al + Cu		3
P4	**Thorax low dose**	35	120	Al + Cu	10–700	0.4

The x‐ray spectra corresponding to each protocol were modeled using the SpekPy software.^42^, ^43^ ^⁠^ The program calculates effective energy, $E_{eff}$, corresponding to each protocol, and these effective energies were used to estimate the linear attenuation coefficient, $\mu (E_{eff})$, at these energies for each polymer and 3D printing material under consideration.

The linear attenuation coefficients of the conventional polymers (polypropylene, PMMA, nylon, and polyurethane) were calculated using the NIST/XCOM database.^44^ ^⁠^ These calculations used the chemical formulae of each material and measured or published mass densities. Additionally, the linear attenuation coefficients of the 3D printing materials (HIPS, VeroClear, VeroBlue, and TangoBlack+) were experimentally determined. For this purpose, samples of the different commercial materials were printed as 5 × 5 cm^2^ blocks with thicknesses between 1 and 2 cm. These samples were used for the experimental evaluation of the linear attenuation coefficients according to the exponential attenuation law using a poly‐energetic narrow beam at energies between 15 and 150 keV^⁠^.^45^, ^46^ All x‐ray measurements were performed using an MCN 421 x‐ray tube (Philips Inc., Netherlands) with a stationary tungsten anode. A cadmium telluride (CdTe) spectrometer (model XR‐100T, Amptek Inc., USA) with an active volume of 3 × 3 × 1 mm^3^ and the PX4 digital pulse processor were used. A more detailed description of the experimental setup, detection system alignment, calibration procedures, and spectral distortion corrections can be found in previous publications.^45^, ^47^ ^⁠^

The HU values of the studied materials were calculated using measured or NIST/XCOM calculated linear attenuation coefficient of the material under consideration (polymer or resin) at the effective energy. The estimated HU values were preliminarily compared to the clinically expected values for the anatomical parts to be simulated by the phantom. These comparisons were useful for pre‐classification of the selected materials deemed adequate for simulating different lung nodules (solid, sub‐solid, ground‐glass, etc.) and the 3D printed tracheobronchial tree.

The final characterization of the materials used on the Freddie‐Mercury construction was performed considering the relative difference between the nominal and the measured HU values, obtained from the phantom images, considering the CT protocols used for testing the prototype (Table 2). HU values were determined using the imQuest software (Duke University, USA). A circular region of interest (ROI) with an area of about 50 mm^2^ was selected for each material. Ten measurements were performed for each material at different positions, and the mean and standard deviation were calculated. The synthetic lung parenchyma was also evaluated by measuring the average HU and estimating its density.

#### 2.4.2 Freddie validation using CT images

**FIGURE 8:**
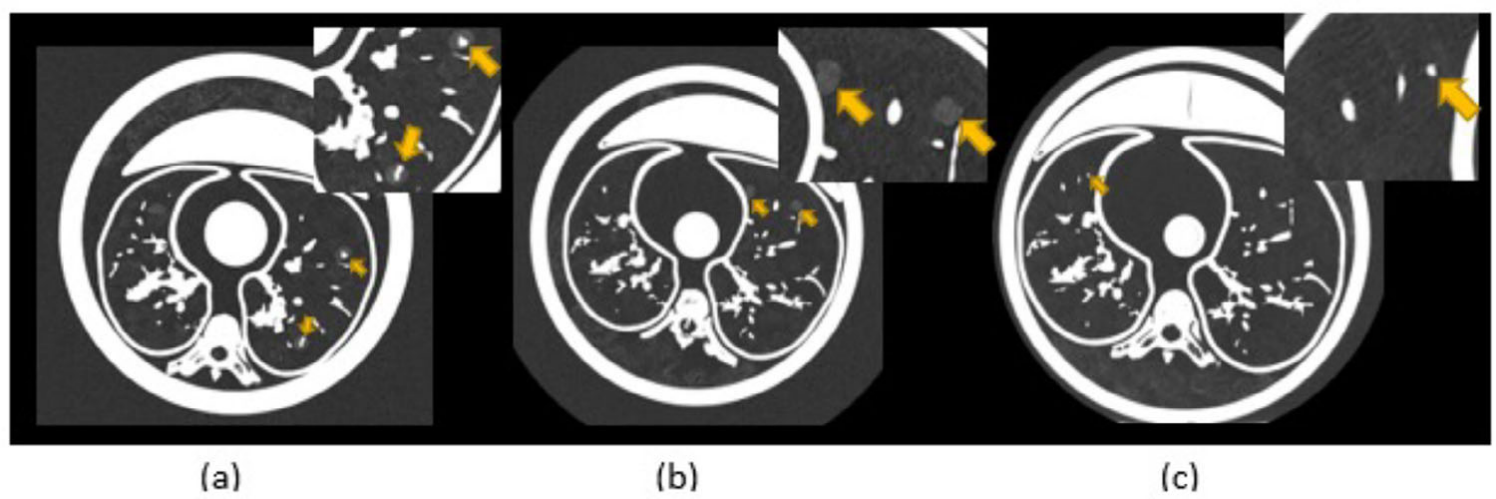
Examples of images of synthetic nodules into the Freddie setup: (a) Two sub‐solid nodules allocated at the right lobule of section 3 (230 mm); (b) Two GGOs allocated at the right lobule of section 2 (300 mm); and (c) one solid nodule allocated at the left lobule of section 1 (370 mm). GGOs, ground‐glass opacities.

The validation of the Freddie phantom was performed using a simplified reader study. The main goal of this study was to get feedback from radiologists and non‐radiologists in order to assess how the synthetic nodules appear, when compared to real nodules. The synthetic nodules of different materials and sizes representing solid, sub‐solid, and ground‐glass opacities previously described were placed in each lung quadrant of the Freddie setup (upper‐right, lower‐right, upper‐left, and lower‐left) of all diameters. The free space was filled with the fractioned packing chips in order to simulate the lung parenchyma. Figure 8 presents examples of resulting images of these nodules imaged in different phantom diameters.

The reader study was designed using the Radboudumc Grand Challenge system^2^. During the study, images of the Freddie setup acquired with the protocols presented in Table 2 were displayed, with the observer able to scroll while searching for lung nodules or other patient‐like structures. The study was not blinded, since each reader received instructions regarding the position of the nodules and their identification codes. The reader could also classify the similarity of the synthetic nodules in comparison to a hypothetical real patient, classifying each finding on a 1–5 Likert scale. Five chest radiologists (3–17 years of experience) and eight non‐radiologists (clinical medical physicists, medical physics residents, and grad and undergrad students) participated in the study. The non‐radiologist readers received additional information regarding the appearance of real (patient) nodules in order to guide their scores. All nodules inserted into the Freddie setup and imaged according to the protocols presented in Table 2 were reviewed by the chest radiologists and non‐radiologist readers.

The same software adopted to calculate nodule volumes using µCT images (3D Slicer) was used to segment and calculate the volume of the nodules imaged using the protocols presented in Table 2. Semi‐automatic segmentation based on region growing was adopted. The estimated volumes were used to determine the accuracy of the volume measurements by calculating the percent relative error (RE) against the volumes determined from µCT images (ground truth). The percent coefficient of variation (CV) was calculated to estimate the precision of the volume measurements.

#### 2.4.3 Task‐based metrics characterization

The task‐based image quality metrics TTF and NPS were determined using the Mercury setup of the developed phantom. The evaluation of the image quality metrics in the studied clinical protocols was performed using the ImQuest software version 7.2 (Duke University, USA). For a specific clinical task and dose, the ImQuest software can be used to determine TTF, NPS, and detectability index. In the present work, the software was set to use an NPWE (non‐prewhitening with the Eckstein eye filter) observer model with a designer‐contrast profile, which is dependent on the peak contrast of the signal against the background. This evaluation serves only to illustrate the applicability of the phantom in this alternative configuration and does not have the aim of evaluating IQ for the protocols or the specific CT system used in this validation experiment.

## 3 RESULTS

**FIGURE 9:**
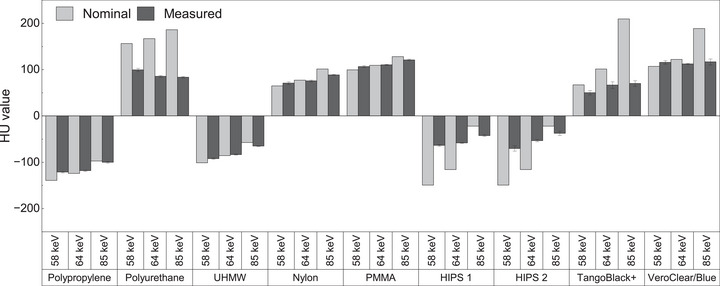
Measured and nominal HU values of the materials used for phantom construction. HU, Hounsfield units.

The effective energies of the P1, P2, and P3/P4 protocols were 58 keV, 85 keV and 64 keV, respectively. Figure 9 summarizes the results of nominal and measured HU values of the materials used for the various protocols. The background lung parenchyma exhibited a value of −909 ± 30 HU. This indicates a density of 0.09 ± 0.03 g/cm^3^.

**FIGURE 10:**
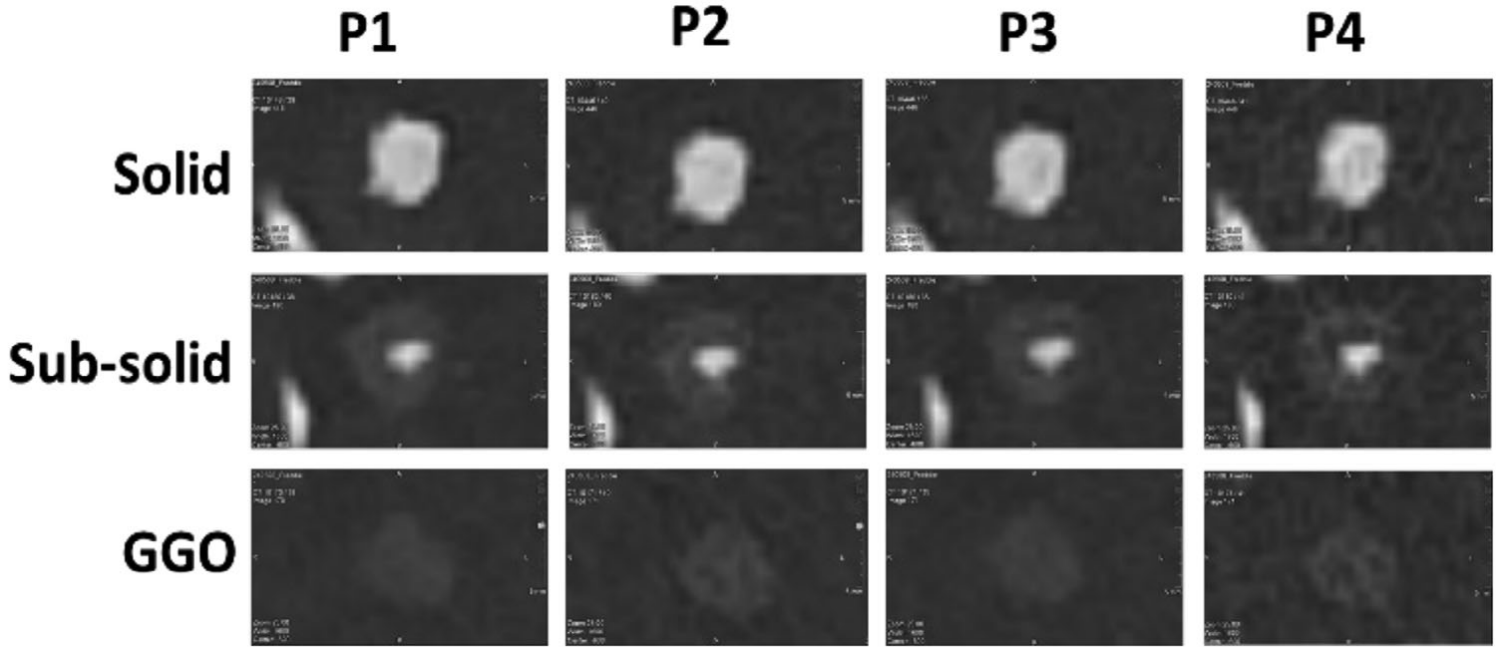
Examples of images of the synthetic lung nodules designed to simulate solid, sub‐solid, and GGOs imaged with the four clinical protocols described in Table 2. GGOs, ground‐glass opacities.

**FIGURE 11:**
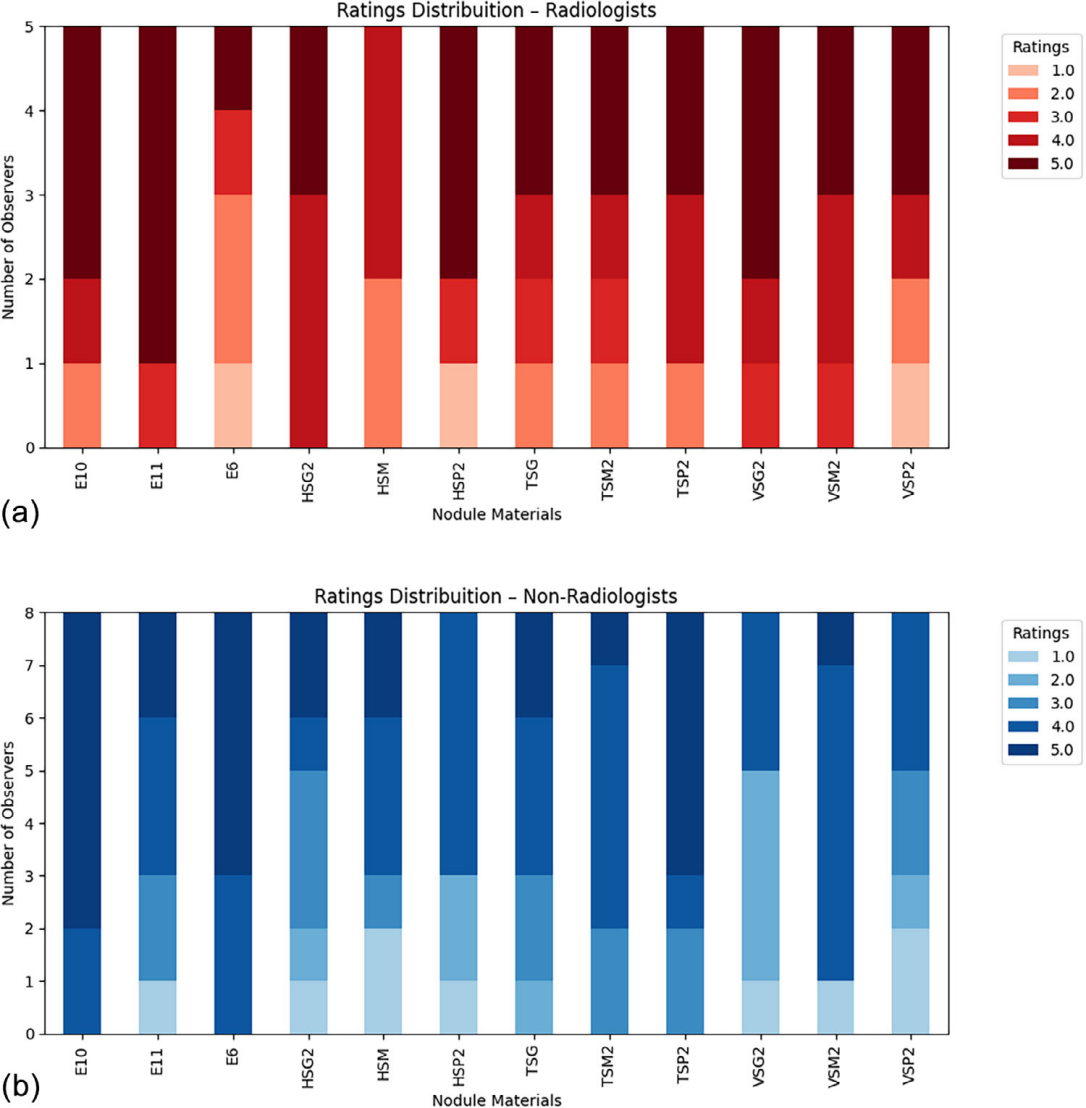
Results of the simplified reader evaluation of the materials for nodule characterization using a Likert scale. HIPS (HSG2, HSM, and HSP2), TangoBlack+ (TSG, TSM2, and TSP2)) and VeroClear (VSG2, VSM2, and VSP2) are materials used for mimicking solid and sub‐solid nodules, and EVA (E6, E10, and E11) was used for mimicking GGOs. Thirteen readers participated in the reading process: (a) 5 radiologists and (b) 8 non‐radiologists. GGOs, ground‐glass opacities; HIPS, high‐impact polystyrene.

The synthetic nodules volumes, with respective uncertainties, are presented in Table 3. The accuracy (RE) and precision (CV) of the volumetry estimation demonstrate strong dependence on the size of the nodule and also on its characteristics (solid or GGO), as expected. Solid nodules volumetry resulted in 0.02% ≤ RE ≤ 25% and 0.4% ≤ CV ≤ 12% while GGO nodules resulted in 0.07% ≤ RE ≤ 18% and 0.7% ≤ CV ≤ 8%. Smaller nodules present large RE and CV, while large nodules result in smaller RE and CV. Figure 10 presents examples of synthetic nodule images resulting from the different clinical protocols. The reader study resulted in that the synthetic solid nodules printed in VeroClear received average Likert scores 4.0 (range 3.0–4.0) from radiologists and 3 (range 2.6–3.8) from non‐radiologists, printed in TangoBlack+ received an average Likert score of 3.9 (range 3.8–4.2) from radiologists and 4.0 (range 3.8–4.4) from non‐radiologists, while those printed in HIPS scored an average Likert of 3.8 (range 3.3–3.9) from radiologists and 3.3 (range 3.1–3.3) from non‐radiologists. The synthetic GGO nodules manufactured in EVA received an average Likert score of 3.8 (range 2.8–4.6) from radiologists and 4.3 (range 3.6–4.8) from non‐radiologists. Figure 11 shows a plot representing the results of this simplified reader study.

**TABLE 3:** Volume of the synthetic nodules calculated using the 3D Slicer software from the µCT images. Photos with examples of these nodules are also presented.

Printed nodules size classif.	Nodule	μ‐CT volume (mm^3^)	Vero Clear nodules examples	Nodule	μ‐CT volume (mm^3^)	TangoBlack nodules examples	Nodule	μ‐CT volume (mm^3^)	HIPS nodule examples	Handmade Nodules size classif.	Nodule	μ‐CT volume (mm^3^)	EVA nodules examples
	VeroClear			TangoBlack			HIPS				EVA		
**XXL**	VC1	6736 ± 146	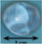	TB1	6533 ± 157	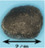	H1	6505 ± 216	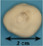	**XL**	E1	2496 ± 96	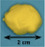
**XL**	VC2	1844 ± 77	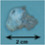	TB2	1787 ± 80	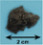	H2	1848 ± 97	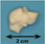	**L**	E2	1412 ± 90	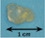
**L**	VC3	1205 ± 60		TB3	1169 ± 63		H3	1161 ± 72					
**M**	VC4	152 ± 14	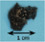	TB4	152 ± 17	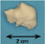	H4	189 ± 22	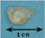		E3	1037 ± 72	
	VC5	154 ± 16		TB5	152 ± 16								
							H5	190 ± 23			E4	1143 ± 68	
	VC6	153 ± 16		TB6	153 ± 16								
**S**	VC7	72 ± 9	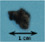	TB7	64 ± 10	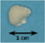	H6	63 ± 11					
									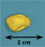		E5	808 ± 49	
	VC8	72 ± 9		TB8	66 ± 9								
							H7	70 ± 11			E6	1299 ± 62	
	VC9	81 ± 10		TB9	65 ± 9								
**XS**	VC10	26 ± 4	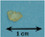	TB10	23 ± 6	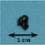	H8	22 ± 6	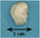		E7	1341 ± 68	
				TB11	23 ± 5								
	
	VC11	26 ± 5									E8	993 ± 52	
							H9	19 ± 5					
				TB12	22 ± 5								
	VC12	27 ± 4											
**XXS**				TB13	4 ± 1	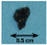	H10	10 ± 4	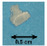	**M**	E9	369 ± 33	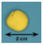
							H11	6 ± 2			E10	428 ± 41	

**FIGURE 12:**
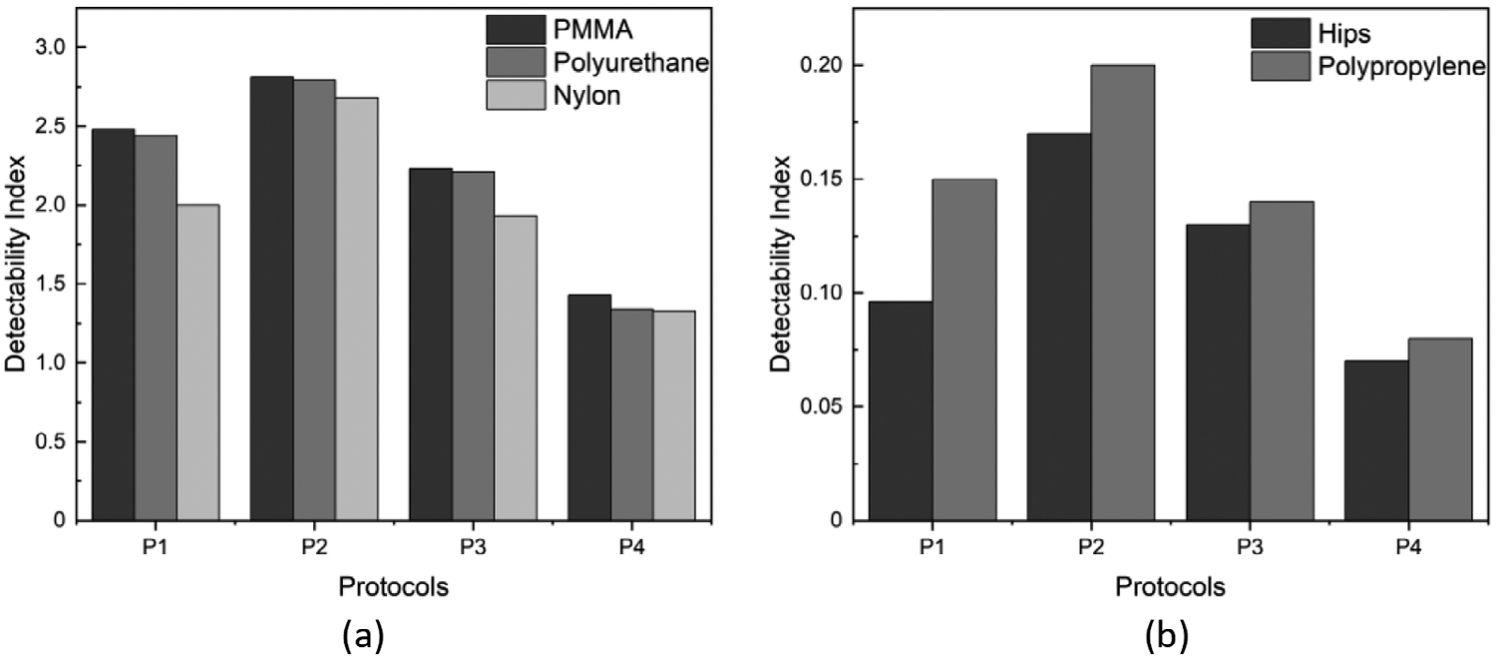
The detectability index data of the 370 mm section of the Mercury phantom for the P1, P2, P3, and P4 protocols. (a) presents the detectability index evaluated using the high contrast inserts (PMMA, polyurethane, nylon), and (b) presents the detectability index determined using the low‐contrast inserts for different materials (polypropylene and HIPS). HIPS, high‐impact polystyrene; PMMA, polymethyl methacrylate.

The detectability indices for each studied protocol were computed using these TTF and NPS functions for each insert material. The results are presented in Figure 12.

## 4 DISCUSSION

In this work, we built a hybrid phantom for evaluating lung CT acquisitions. The materials used to construct the task‐based metrics setup are based on the well‐known Mercury phantom, with a variety of inserts designed to assess a representative range of contrasts found in chest images. As previously emphasized, the goal of the anthropomorphic setup is not to accurately mimic the chest anatomy, but to represent attenuation properties similar to different effective‐diameter patients. This design enables the evaluation of the automatic TCM response of the CT systems in chest‐like structures. Successful examples of the application of the developed phantom are presented, exploring the effect of different protocols and dose levels on the identification of lung nodules with different sizes and morphologies. The effectiveness of this approach was demonstrated by the scores given by experienced observers to the images obtained with these nodules.

The agreement between nominal and observed HU values in the physical setup was within 15%, except for TangoBlack+ across all three effective energies and in HIPS and VeroClear/Blue at 85 keV. These discrepancies may stem from inherent variations in material composition, or slight deviations in the actual chemical composition or mass density compared to the idealized composition assumed in NIST/XCOM. Also, the microstructural inconsistencies introduced during the 3D printing process may cause differences. Furthermore, the infill percentage of the printed materials, especially those produced using the fused deposition modeling technique, might impact the final density and, consequently, the measured HU values⁠.^48^

As expected, the use of the Mercury setup in combination with the imQuest software successfully demonstrated detectability index variation between the studied protocols, influenced by the dose levels, voltage, and x‐ray beam filtration used. Materials such as PMMA, polyurethane, and nylon presented higher detectability values, while the polypropylene and HIPS materials showed lower detectability. This results from the difference in the contrast of these materials against the UHMW polypropylene background. The detectability variation between protocols is more noticeable for high‐contrast materials than for low‐contrast materials.

The actual synthetic nodule volume accurately represents volumes typically observed in clinical settings and serves as a reliable reference for reading studies utilizing the developed anthropomorphic setup. These studies can be configured in a variety of formats, enabling a comprehensive exploration of different nodule sizes, radiodensities, and patient dimensions, given their compatibility with all four diameters of the Freddie setup. These advantages can be explored in studies considering different nodules' properties (radiomic signatures) and volumetry.^49^ ^⁠^ Additionally, future developments may allow for investigation of other lung disease detectability or other anatomies of clinical interest.

The combined response of the application of the Freddie‐Mercury phantom across four protocols on a single CT system demonstrates the association between the detectability index and the visual characteristics of the synthetic lung nodules. For example, the P2 protocol yielded higher values of the detectability index and resulted in nodule images with lower noise and higher spatial resolution (Figure 10). On the other side, LDCT images (P4 protocol) presented lower values of the detectability index and noisier images, which may result in a potential loss of GGOs visibility.

The 40% contrast difference criterion adopted when comparing phantom to patient in the same anatomical region was chosen to reflect the current state of available 3D printing materials and their limitations in accurately replicating the attenuation properties of human tissues. Although an advance in 3D printing technologies and materials, there are still challenges in achieving exact material properties that match the complex attenuation behavior of biological tissues, particularly in the chest region, where subtle contrast variations are crucial. Variability in material composition, polymerization processes, fillers, and printing resolution can lead to differences in linear attenuation coefficients, making it difficult to match HU values precisely. The 40% criterion represents a balance, ensuring the selected materials provide phantoms with contrast levels close to human tissue, without unnecessarily narrowing the range of usable materials.

The analysis of the similarity between synthetic nodules printed with different materials and the appearance of real nodules in tomographic images was conducted through a simplified observational study involving five radiologists and eight non‐radiologists. The radiologists identified VeroClear and TangoBlack+ as the materials that best resemble real nodules, whereas the non‐radiologists considered TangoBlack+ to be the material most similar to real nodules.

As a prototype, the presented device has limitations. For example, obvious anatomical features of chest CT images were not included, such as the heart, major vessels, muscles, and ribs. Depending on the application, if these additional anatomical structures are shown to be essential for an accurate evaluation of the anthropomorphic setup, they could be incorporated in future versions.

## 5 CONCLUSIONS

This work presents the design and validation of a hybrid phantom composed of two setups, one for task‐based image quality metrics and one anthropomorphic. The novelty of the proposed design is concentrated on the possibility of associating the response of the task‐based metrics setup (Mercury) with a patient‐based setup (Freddie) in a single phantom. This hybrid design enhances the potential to apply the detectability index for supporting protocol optimization, considering clinical scenarios.

## CONFLICT OF INTEREST STATEMENT

The authors declare no conflicts of interest.

## Footnotes

1 The study was approved by the Institutional Board Review (CAAE 27912619.6.0000.0068) of the Hospital das Clínicas, Faculdade de Medicina, Universidade de São Paulo (HCFMUSP), São Paulo, Brazil, and informed consent was not required.

^2^
The Radboudumc Grand Challenge is a platform for end‐to‐end development of machine learning solutions in biomedical imaging (https://grand‐challenge.org/)
